# HIV drug resistance and its associated factors among patients during interruption of antiretroviral therapy in China

**DOI:** 10.3389/fmicb.2025.1617795

**Published:** 2025-07-11

**Authors:** Zhongbao Zuo, Wei Huang, Lei Liu, Miaomiao Li, Hongli Chen, Yi Feng, Lingjie Liao, Yiming Shao, Yuhua Ruan, Jing Wu, Hui Xing

**Affiliations:** ^1^Department of Clinical Laboratory, Hangzhou Xixi Hospital, Zhejiang, China; ^2^Department of Orthopedics, Hangzhou Xixi Hospital, Zhejiang, China; ^3^National Center for AIDS/STD Control and Prevention, Chinese Center for Disease Control and Prevention, Beijing, China

**Keywords:** HIV, drug resistance, interruption of antiretroviral therapy, mutation, viral load

## Abstract

**Background:**

The prevalence of human immunodeficiency virus (HIV) drug resistance among people living with HIV (PLWH) who experience treatment interruptions is a significant concern. This study aimed to investigate the prevalence and characteristics of HIV drug resistance for PLWH who experienced treatment interruptions in China.

**Methods:**

This study included 595 PLWH from four studies conducted in China between 2003 and 2021. Data were collected through face-to-face questionnaires, and HIV drug resistance was genotyped using an in-house assay. Multivariate logistic regression analysis was performed to identify factors associated with drug resistance.

**Results:**

The prevalence of drug resistance was 19.6% (83/424). The most frequent drug resistance mutation was K103N (12.9%). The HIV drug resistance rate and the frequency of patients harboring ≥2 drug resistance mutations decreased significantly in the longer interruption time group. Chi-square trend tests showed that HIV drug resistance, NNRTI drug resistance, K1O3N mutation, and viral load significantly decreased in the longer interruption time group. Multivariate logistic regression analysis revealed that viral load ≥15,000 copies/mL (AOR: 0.58, 95% CI: 0.34–0.98, *p* = 0.04), interruption time ≥24 months (AOR: 0.29, 95% CI: 0.13–0.63, *p* = 0.002), and ART duration of 12–24 months before interruption (AOR: 0.36, 95% CI: 0.17–0.76, *p* = 0.01) were significantly associated with HIV drug resistance.

**Conclusion:**

The study found that the prevalence of HIV drug resistance among PLWH who experienced treatment interruptions in China was 19.6%. These findings highlight the importance of conducting drug resistance tests for patients with interruptions before reinitiating ART, especially when the viral load is> 1,000 copies/mL.

## 1 Introduction

The overall incidence trend of human immunodeficiency virus/acquired immunodeficiency syndrome (HIV/AIDS) in China has been stable in recent years (Yang et al., 2024; Ye et al., 2024). By June 30, 2024, China had reported 1,329,127 cases of people living with HIV/AIDS (The Chinese Center for Disease Control and Prevention Sexually Transmitted Disease of AIDS Prevention and Control Center, 2024). China’s National Free Antiretroviral Treatment Program (NFATP) started earlier in 2002 with limited antiretroviral therapy patients and then adopted the “test and treat” strategy in 2016, where patients were included in antiretroviral therapy (ART) regardless of their CD4 + T cell counts (Wang et al., 2024). ART has significantly improved the quality of life and survival rate for people living with HIV (PLWH) in China (Wang et al., 2024). However, with the expansion of ART, the number of PLWH who stopped ART also increased. The interruption rates of antiretroviral therapy for PLWH in Guangxi, China, from 2010 to 2018 (Jia et al., 2022), and Chongqing, China, from 2013 to 2017 (Zhou et al., 2020) were 5.52/100 person-years and 4.6/100 person-years, respectively. During China’s COVID-19 nonpharmaceutical interventions, numerous HIV patients ceased treatment (Sun et al., 2020). However, whether these patients re-initiate treatment and their drug-resistance status remains unclear. The interruption rate in Eritrea (Mengistu et al., 2022) between 2005 and 2020 was 3.2 events/100 person-years, and previous studies (Touré et al., 2023; Munyayi and van Wyk, 2023) found that the rate of treatment interruption at 36 months ranges from 15.4 to 26.0%. A study (Mtisi et al., 2023) in Tanzania found that 26.2% (546/2084) of adolescents followed for 2 years had interrupted treatment. However, a comprehensive model of HIV transmission dynamics (Shen et al., 2023) has demonstrated that even a slight increase in interruption rates, in the range of 0.0114 to 0.0220, is sufficient to offset the benefits of treatment expansion and exacerbate the transmission dynamics of HIV drug resistance. Therefore, managing patients who have interrupted ART and capturing the virological, immunological, and drug resistance characteristics of drug withdrawal patients is crucial.

HIV drug resistance can lead to the failure of antiviral therapy and a significant increase in the risk of death (Li et al., 2023; Zhang et al., 2021). Studies in Malawi (Luebbert et al., 2012) and China (Li et al., 2021) found that 24.1% (32/133) and 19.5% (34/174) of PLWH with interrupted treatment exhibited drug resistance to the first-line regimen. Viral fitness, the ability of a virus to produce adaptive and rapidly replicating offspring, is inversely correlated with antiretroviral resistance mutations. Without antiretroviral drugs, the wild-type virus with fewer resistance mutations can typically outcompete and outgrow more resistant strains (Li et al., 2021; Wang et al., 2011; Lawrence et al., 2003). An analytical treatment interruption study (Hunter et al., 2021) found that 57% of mutations in plasma reverted to wild type at week 12, and the authors estimated that the mean weeks until complete reversion to wild type for Protease Inhibitor (PI), Nucleoside Reverse Transcriptase Inhibitor (NRTI), and Non-Nucleoside Reverse Transcriptase Inhibitor (NNRTI) were 33.7, 20.9, and 19.8 weeks, respectively. However, some research studies were restricted by their limited sample size or shorter observation time.

Currently, studies on drug-resistant HIV patients who stopped treatment are scarce. This is primarily attributed to the challenges in tracking patients who have interrupted treatment and the significant human and material resources required for such studies. Existing studies are fraught with limitations, including small sample sizes (Wang et al., 2011; Vazquez-Guillen et al., 2016), and most were initiated over a decade ago (Wang et al., 2011; Lawrence et al., 2003). Furthermore, many of these studies involved structured treatment interruptions (Hunter et al., 2021; Vazquez-Guillen et al., 2016). This study sought to investigate HIV drug resistance among PLWH who experienced treatment interruptions in China, as well as the factors associated with HIV drug resistance. Additionally, the study examined the characteristics of changes in HIV drug resistance mutations among patients who discontinued antiretroviral therapy.

## 2 Methods

### 2.1 Study design and participants

**Figure 1:**
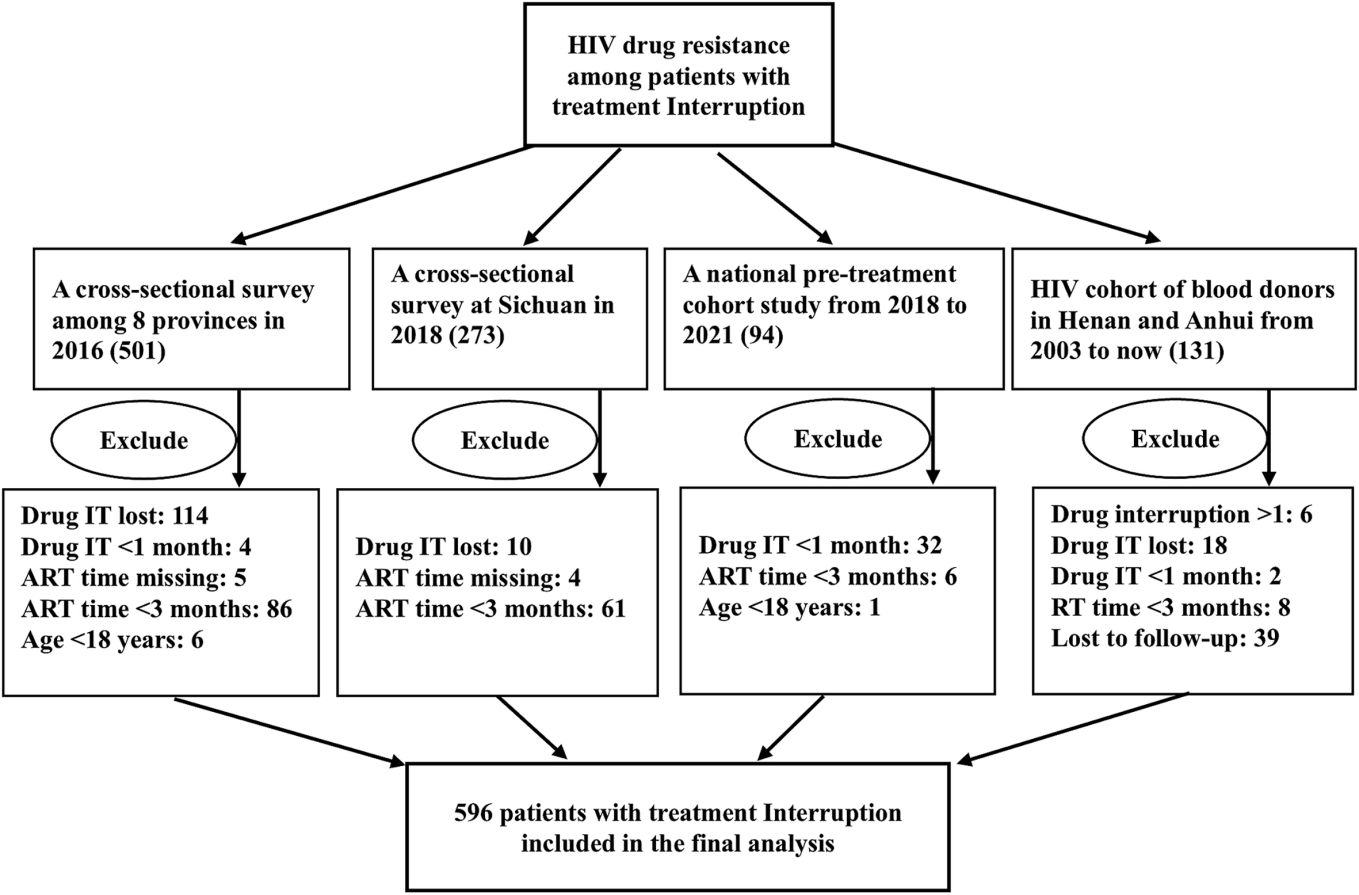
Flowchart of HIV patients with treatment interruption and patient enrollment, 596 patients were included in the final analysis. IT, interruption time.

This study encompassed four studies conducted in China from 2003 to 2021: (1) A cross-sectional survey among 8 provinces in 2016; (2) A cross-sectional survey in Sichuan in 2018; (3) A national pre-treatment cohort study from 2018 to 2021; and (4) HIV cohort of blood donors in Henan and Anhui from 2003 to 2021. The exclusion criteria were: (1) drug interruption time missing or < 1 month; (2) ART duration before interruption missing or < 3 months; (3) age < 18 years old; and (4) unwilling to participate in this study or provide written informed consent. The study flowchart is presented in Figure 1. The plasma samples of PLWH were transported to the laboratory at the National Center for AIDS/STD Control and Prevention (NCAIDS), Chinese Center for Disease Control and Prevention (China CDC), where the HIV drug resistance was genotyped. This research was approved by the NCAIDS, China CDC (X140617334). All data utilized in this study were anonymized to ensure confidentiality.

### 2.2 Data collection

The data were collected through a face-to-face questionnaire by the trained local medical staff. The demographic information, ART interruption time, and ART duration before interruption were collected during the interview. Post-interview blood samples were obtained. CD4 + T lymphocytes were enumerated via flow cytometry at the local CDC within 12 h. The plasma was subsequently separated and transported to the NCAIDS, China CDC. For samples with a viral load (VL) of 1,000 copies/mL or higher, HIV drug resistance genotyping was conducted using an in-house assay (Inzaule et al., 2013; Zuo et al., 2016). HIV drug resistance mutation was identified and interpreted using the algorithm from the Stanford HIV Drug Resistance Database.^1^ HIV drug resistance was categorized as those conferring low, intermediate, or high levels of resistance (Kang et al., 2020; Chen et al., 2023).

### 2.3 Variable definition and grouping

The median VL in 424 HIV patients was 14,972 copies/mL, so we used a cutoff of 15,000 copies/mL in the following analysis. The education variable had five levels (illiterate, primary school, junior high school, high school, and college degree or above) and was classified into two groups (primary school or below, junior high school or above). The job variable had six levels (farmer, go out to work, business or self-employed, enterprise or company, organs or institutions, and others) and was classified into two groups (farmer, others). The marriage variable had three levels (married or cohabiting, unmarried, and others). The route variable had four levels (heterosexual, homosexual, drug abuse, and others) and was classified into three groups (sexual transmission, drug abuse, and others). The economic variable had four groups (poor, average economic condition, well off, and rich) and was classified into two groups (poor, average economic condition or above). The social support variable had four levels (always, often, sometimes, and never). The self-satisfaction variable had four levels (very satisfied, satisfied, unsatisfied, and very unsatisfied) and was classified into two groups (satisfied, unsatisfied). The alcohol variable had six levels (never drink, once a month or less, 2–3 times a month, 1–3 times a week, 4–6 times a week, and every day) and was classified into two groups (never drink, drink).

### 2.4 Statistical analysis

Numerical variables are presented as mean (standard deviation, SD) or medians (interquartile ranges, IQR) as required. Categorical variables are presented as proportions. Evaluations of the representativeness of 424 successfully genotyped, 595 included, and the 999 HIV patients were conducted in our study. For categorical variables such as gender, we use the chi-square test to compare whether there is a statistical difference among the three groups; For continuous variables such as age, we use analysis of variance (ANOVA) to compare whether there is a statistical difference between the three groups. The interruption time was categorized into five levels (1–6 months, 6–12 months, 12–18 months, 18–24 months, and ≥24 months). Using R package “CATT” to perform trend chi-square tests among the five interruption time groups for HIV drug resistance, NNRTI drug resistance, K103N, NRTI drug resistance, CD4 + T cells, and viral load. Univariate logistic regression analyses were performed to identify potential factors associated with drug resistance. Subsequently, a stepwise multivariate logistic regression model was employed to select variables that were independently associated with drug resistance. Two-sided *p*-values <0.05 were considered statistically significant in our study. All statistical analyses were conducted with R software (version 4.0.2, R Development Core Team 2020).

## 3 Results

### 3.1 Characteristics of PLWH interrupted with ART

A total of 595 PLWH were included in the final analysis (Table 1). The median age of the PLWH was 40.0 years (IQR: 33.0–50.5); 32.3% (192/595) were female, 50.4% (300/595) were Han, and 66.7% (397/595) had a primary school education or below. Additionally, 77.5% (461/595) lived in rural areas, 70.3% (418/595) were farmers, and 67.6% (402/595) were married or cohabiting at the time of the survey. In terms of transmission routes, 44.4% (264/595) were infected through heterosexual contact, 4.2% (25/595) through homosexual contact, and 37.3% (222/595) through drug abuse. Finally, 43.0% (256/595) were unsatisfied with their lives. The median CD4 + T cell count at the survey was 313 (IQR: 196–463) cells/μL, and the VL at the survey was 9,218 (IQR: 2100–46,414) copies/mL. The median interruption time was 12.97 (IQR: 5.67–27.27) months, and the median ART duration for PLWH before drug interruption was 19.10 (IQR: 9.95–34.50) months. 86.2% (513/595) were started with the First-line regime, but 71.5% (425/595) were ended with the First-line regime. 424 PLWH were successfully genotyped, and 83 (19.6%) patients were found to have drug resistance. Evaluations of the representativeness of 424 successfully genotyped, 595 patients included, and the original 999 HIV patients were shown in Supplementary Table 1. The result showed that 424 successfully genotyped and 596 patients were representative.

**Table 1:** Characteristics of 595 HIV patients with antiretroviral treatment interruption in this study.

Variable	Overall (%)
*N*	595
Age (median [IQR], years)	40.00 (33.00–50.50)
Gender = Female (%)	192 (32.3)
Ethnic = Han (%)	300 (50.4)
Education	
Primary school or below	397 (66.7)
Junior high school or above	196 (33.0)
Missing	2 (0.3)
Residence registration	
Rural areas	461 (77.5)
Urban areas	74 (12.4)
Missing	60 (10.1)
Job	
Farmer	418 (70.3)
Others	149 (25.0)
Missing	28 (4.7)
Marriage	
Married or cohabiting	402 (67.6)
Unmarried	95 (15.9)
Other	69 (11.6)
Missing	29 (4.9)
Route	
Heterosexual	264 (44.4)
Homosexual	25 (4.2)
Drug abuse	222 (37.3)
Other	84 (14.1)
Spouse or fixed sexual partner infected with HIV	
Yes	165 (27.7)
No	169 (28.4)
No spouse or fixed sexual partner	133 (22.4)
Missing	128 (21.5)
Economy	
Poor	243 (40.8)
Average economic condition or above	233 (39.2)
Missing	119 (20.0)
Social Support	
Always	16 (2.7)
Often	121 (20.3)
Sometimes	299 (50.3)
Never	41 (6.9)
Missing	118 (19.8)
Self-satisfied	
Satisfied	220 (37.0)
Unsatisfied	256 (43.0)
Missing	119 (20.0)
Alcohol	
Never drink	180 (30.3)
Drink	291 (48.9)
Missing	124 (20.8)
CD4 at the survey (median [IQR], cells/ul)	313 [196, 463]
Viral Load at the survey (median [IQR], copies/ml)	9,218 [2,100, 46,414]
Interruption time (median [IQR], months)	12.97 [5.67, 27.27]
ART time (median [IQR], months)	19.10 [9.95, 34.50]
Initial ART regime	
First-line regime	513 (86.2)
Second-line regime	20 (3.4)
Others	16 (2.7)
Missing	46 (7.7)
Stop ART regime	
First-line regime	425 (71.5)
Second-line regime	85 (14.2)
Others	19 (3.2)
Missing	66 (11.1)
HIV Drug Resistance	
No	341 (57.3)
Yes	83 (14.0)
Missing	171 (28.7)

### 3.2 Drug resistance mutations among different interruption times

**Figure 2:**
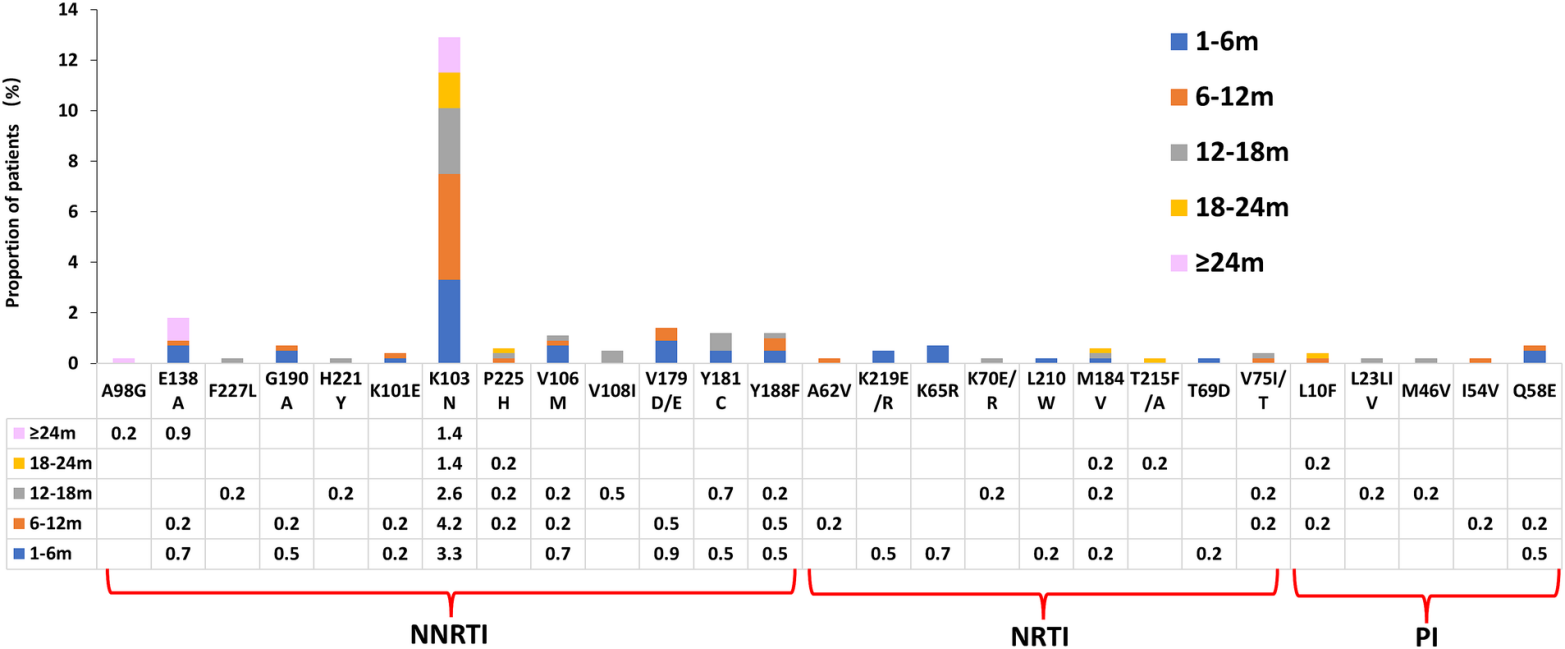
HIV drug resistance mutations among different ART interruption times.

Drug resistance mutations were found at all interruption time intervals (Figure 2). The most frequent drug-resistance mutation was K103N (12.9%, 55/424), followed by E138A (1.8%, 8/424) and V179D/E (1.4%, 6/424). The K103N mutation may affect the virus’s replication ability with little effect, and the K103N mutation significantly differed between the interruption time 1–18 m group (43/242) and the ≥ 18 m group (12/182) (Table 2; Figure 2). PLWH with short drug interruption time exhibited specific mutations, whereas those with longer interruption time showed different mutation patterns. Some drug-resistance mutations, such as G190A, K101E, P225H, V179D/E, Y181C, Y188F, K219E/R, K65R, and Q58E, appeared in PLWH with short drug interruption times, but the mutations disappeared when PLWH had a longer interruption time. 8.2% (8/98) of patients harboring ≥2 drug-resistant mutations in the ART interruption time 1–6 months group, while 4.4% (2/46) and 0% in 18–24 months group and ≥24 months group, respectively (Table 2). The frequency of patients harboring ≥2 drug-resistant mutations significantly differed among the different interruption time groups.

**Table 2:** Chi-square trend test of HIV drug resistance, VL, and CD4 in different ART interruption times.

Variable	ART interruption time					*P* for trend
	1–6m	6–12m	12–18m	18–24m	≥24m	
HIV drug resistance						0.002
No	74 (75.5)	74 (74.0)	29 (65.9)	39 (84.8)	125 (91.9)	
Yes	24 (24.5)	26 (26.0)	15 (34.1)	7 (15.2)	11 (8.1)	
NNRTI drug resistance						0.01
No	78 (79.6)	77 (77.0)	31 (70.5)	40 (87.0)	125 (91.9)	
Yes	20 (20.4)	23 (23.0)	13 (29.5)	6 (13.0)	11 (8.1)	
K103N						0.02
No	84 (85.7)	82 (82.0)	33 (75.0)	40 (87.0)	130 (95.6)	
Yes	14 (14.3)	18 (18.0)	11 (25.0)	6 (13.0)	6 (4.4)	
NRTI drug resistance						0.19
No	94 (95.9)	99 (99.0)	42 (95.5)	44 (95.7)	136 (100.0)	
Yes	4 (4.1)	1 (1.0)	2 (4.5)	2 (4.3)	0 (0.0)	
PI drug resistance						0.15
No	95 (96.9)	97 (97.0)	42 (95.5)	45 (97.8)	136 (100.0)	
Yes	3 (3.1)	3 (3.0)	2 (4.5)	1 (2.2)	0 (0.0)	
VL≥15000 copies/ml						<0.001
No	61 (62.2)	61 (61.0)	20 (45.5)	18 (39.1)	53 (39.0)	
Yes	37 (37.8)	39 (39.0)	24 (54.5)	28 (60.9)	83 (61.0)	
CD4 ≤200 cells/ul^a^						0.44
No	72 (76.6)	74 (74.7)	20 (50.0)	33 (71.7)	93 (71.0)	
Yes	22 (23.4)	25 (25.3)	20 (50.0)	13 (28.3)	38 (29.0)	

a CD4 deletion in 14 patients.

### 3.3 Chi-square trend test of HIV drug resistance, VL, and CD4 in different ART interruption times

The rates of HIV drug resistance were significantly different among different ART interruption time groups, and the P for trend was 0.002 (Table 3). Similarly, NNRTI drug resistance, K103N mutation, and viral load showed trends among different drug interruption times: NNRTI resistance and K103N mutations decreased in the longer interruption time group, while viral load increased. However, NRTI drug resistance, PI drug resistance, and CD4 showed no significant difference among different interruption times.

**Table 3:** Number of drug-resistant mutations among 83 drug-resistant patients at different ART interruption times.

Number of drug-resistant mutations	ART interruption time				
	1–6 m (98)	6–12 m (100)	12–18 m (44)	18–24 m (46)	≥24 m (136)
0	74 (75.5%)	74 (74.0%)	29 (65.9%)	39 (84.8%)	125 (91.9%)
1	16 (16.3%)	21 (21.0%)	9 (20.5)	5 (10.9%)	11 (8.1%)
≥2	8 (8.2%)	5 (5.0%)	6 (13.6%)	2 (4.4%)	
2	4 (4.1%)	4 (4.0%)	3 (6.8%)	1 (2.2%)	
3	2 (2.0%)	1 (1.0%)	1 (2.3%)	1 (2.2%)	
4			2 (4.5%)		
5	1 (1.0%)				
6	1 (1.0%)				
*P*	<0.001				

### 3.4 Multivariable logistic analysis of drug resistance for patients with drug interruption

Among the 424 patients, 83 (19.6%) had at least one drug-resistant mutation. Univariate logistic regression analysis revealed significant differences between HIV drug resistance and job, viral load, interruption time, and ART duration (*p* < 0.05). Significant variables in univariate logistic regression were included in the multivariate logistic regression, and the results showed that viral load ≥15,000 copies/mL (AOR: 0.58, 95% CI: 0.34–0.98, *p* = 0.04) compared with viral load <15,000 copies/mL, interruption time ≥24 months (AOR: 0.29, 95% CI: 0.13–0.63, *p* = 0.002) compared with interruption time 1–6 month, and ART duration 12–24 months (AOR: 0.36, 95% CI: 0.17–0.76, *p* = 0.01) compared with 3–12 months were significantly correlated with HIV drug resistance (Table 4).

**Table 4:** Multivariate logistic regression analysis of HIV drug resistance in HIV/AIDS patients with antiretroviral treatment interruption in China.

Variable	DR rate	OR	*P*	AOR	*P*
Age	19.6% (83/424)	1.01 (0.99–1.02)	0.41		
Gender					
Male	17.8% (49/275)				
Female	22.8% (34/149)	1.36 (0.83–2.22)	0.22		
Ethnic					
Han	22.9% (48/210)				
Minority	16.4% (35/214)	0.66 (0.40–1.07)	0.09		
Education					
Primary school or below	18.7% (57/305)				
Junior high school or above	21.4% (25/117)	1.18 (0.69–1.99)	0.53		
Residence registration					
Rural areas	19.1% (67/351)				
Urban areas	27.5% (14/51)	1.60 (0.80–3.08)	0.17		
Job					
Farmer	17.2% (56/325)				
Others	27.3% (27/99)	1.80 (1.05–3.03)	0.03		
Marriage					
Married or cohabiting	19.0% (59/310)				
Unmarried	27.1% (19/70)	1.58 (0.86–2.85)	0.13		
Other	11.4% (5/44)	0.55 (0.18–1.33)	0.22		
Route					
Sexual transmission	21.2% (39/184)				
Drug abuse	14.5% (24/165)	0.65 (0.37–1.13)	0.13		
Other	27.6% (21/76)	1.46 (0.78–2.68)	0.23		
Spouse or fixed sexual partner infected with HIV					
Yes	14.9% (18/121)				
No	22.5% (27/120)	1.66 (0.87–3.25)	0.13		
No spouse or fixed sexual partner	21.1% (20/95)	1.53 (0.76–3.10)	0.24		
Economy					
Poor	16.5% (29/176)				
Average economic condition or above	20.8% (35/168)	1.33 (0.77–2.31)	0.30		
Social support					
Always or often	20.4% (19/93)				
Sometimes or never	18.3% (46/252)	0.87 (0.48–1.61)	0.65		
Self-satisfied					
Satisfied	19.5% (31/159)				
Unsatisfied	18.4% (34/185)	0.93 (0.54–1.60)	0.79		
Alcohol					
Never drink	19.2% (25/130)				
Drink	18.5% (39/211)	0.95 (0.55–1.68)	0.86		
CD4 at survey (cells/ul)					
<200	22.9% (27/118)				
≥200	17.1% (50/292)	0.70 (0.41–1.19)	0.18		
Viral load at survey (copies/ml)					
<15000	24.9% (53/213)				
≥15000	14.2% (30/211)	0.50 (0.30–0.82)	0.01	0.58 (0.34–0.98)	0.04
Interruption time (months)					
1–6	24.5% (24/98)				
6–12	26.0% (26/100)	1.08 (0.57––2.07)	0.81	1.04 (0.54–2.02)	0.90
12–24	24.4% (22/90)	1.00 (0.51–1.94)	0.99	1.13 (0.56–2.26)	0.73
≥24	8.1% (11/136)	0.27 (0.12–0.57)	<0.001	0.29 (0.13–0.63)	0.002
ART time (months)					
3–12	35.5% (27/135)				
12–24	10.3% (12/116)	0.46 (0.22–0.94)	0.04	0.36 (0.17–0.76)	0.01
24–36	26.3% (20/76)	1.43 (0.73–2.76)	0.29	1.12 (0.51–2.09)	0.90
≥36	24.7% (24/97)	1.32 (0.70–2.46)	0.39	0.88 (0.48–1.82)	0.84
Initial ART regime					
First-line regime	19.8% (74/374)				
Others	22.7% (5/22)	1.19 (0.38–3.13)	0.74		
Stop ART regime					
First-line regime	18.6% (58/311)				
Others	25.0% (21/84)	1.45 (0.81–2.54)	0.20		

## 4 Discussion

This study, derived from four national surveys in China, included 595 PLWH who had interrupted ART, with 424 gene sequences successfully amplified. The study aimed to examine the prevalence and characteristics of HIV drug resistance among PLWH who experienced treatment interruptions and their associated influencing factors in China. Our study found that the prevalence of drug resistance in China was 19.6% (83/424). Both the HIV resistance rate and the frequency of patients with ≥2 drug resistance mutations decreased significantly in the longer interruption time group. Viral load, drug interruption time, and ART duration before drug interruption were correlated considerably with HIV resistance for PLWH.

Our study found that the prevalence of HIV drug resistance in China was 19.6% (83/424). A study in Spain (Sued et al., 2015) reported that after four structured treatment interruption cycles, only one of twelve patients developed a K70R resistance mutation. However, the unplanned treatment interruption showed a different performance. 27.4% (17/62) patients (Iarikov et al., 2010) from the USA off ART for at least 2 months had drug resistance mutations, but research in Brazil (Kalmar et al., 2012) using HIV DNA from the peripheral blood mononuclear cells (PBMCs) found that 27.7% (38/137) of patients harbored a drug-resistant strain. 64.7% (11/17) of patients in Kenya (Mann et al., 2013) who withdrew from ART for at least 1 week had drug-resistance mutations. The primary purpose of structured treatment interruption is to evaluate the recovery of immune function, reduce drug side effects, and optimize treatment scheme. Therefore, limited studies focus on HIV drug resistance, and the rate of drug resistance is relatively low. However, the drug resistance rate associated with unplanned interruptions in other countries was higher than that in China. One possible explanation for the lower drug resistance rate in China is the longer interruption times observed in our study population. 32.1% (136/424) of patients had interruption times exceeding 24 months. Brazil (Kalmar et al., 2012) and Kenya (Mann et al., 2013) did not include patients who had interrupted ART for more than 24 months, and the USA (Iarikov et al., 2010) only included a small portion of patients who had interrupted ART for more than 24 months, which is also smaller than our study (Iarikov et al., 2010; Kalmar et al., 2012; Mann et al., 2013).

Compared with patients whose VL < 15,000 copies/mL, those with VL ≥ 15,000 copies/mL were less likely to have drug resistance. Generally, patients with high viral load had longer drug withdrawal periods, so the resistant strains in their bodies will gradually be replaced by wild-type strains. Although other studies (Iarikov et al., 2010; Kalmar et al., 2012) found that patients in the resistant group had higher viral loads compared to those in the non-resistant group, the *p* values were not significant. This may be attributed to our study having a larger sample size and a longer observation period. Similarly, compared with patients who had interruption times 1–6 6 months, those with interruption time ≥24 months were also less likely to have drug resistance (AOR: 0.34, 95% CI: 0.15–0.77, *p* = 0.01).

It has been recognized that without the pressure of antiretroviral drugs, drug-resistant mutations will soon be overgrown by wild-type viruses (Wang et al., 2011). The HIV resistance rate, NNRTI drug resistance, K103N mutation, viral load, and the frequency of patients harboring ≥ 2 drug resistance mutations decreased in the longer interruption time group. A study (Wang et al., 2011) focused on the evolution of HIV drug-resistant mutations during ART interruption found that the multiple drug-resistant virus fitness < single drug-resistant virus fitness < wild-type virus fitness. Therefore, the frequency of patients with ≥2 drug-resistant mutations would decrease significantly in the longer interruption time group. However, we still found that the drug resistance mutations existed even in patients longer than 24 months. NNRTI mutations do not impair the fitness of HIV-1 as NRTI and PIs mutations do (Hunter et al., 2021; Kühnert et al., 2018), so NNRTI mutations are expected to last longer. The highly transmitted NNRTI drug resistance in China (Chen et al., 2023) proved that the NNRTI mutation does not impair the fitness cost of viruses. Also, the study (Kühnert et al., 2018) estimated that K103N had a transmission ratio of 0.97 (95% CI: 0.57–1.41), implying that the mutation has no fitness disadvantage or advantage. Therefore, the drug resistance rate of PLWH will decline along with the interruption time, but some patients still carry drug resistance mutations. However, according to the current Chinese guideline (Acquired Immunodeficiency Syndrome Professional Group, Society of Infectious Diseases, Chinese Medical Association, Chinese Center for Disease Control and Prevention, 2024), HIV drug resistance testing is recommended for patients who have stopped ART within 4 weeks. European AIDS Clinical Society (EACS) (EACS, 2024) and U.S Department of Health and Human Services (DHHS) (DHHS, 2024) guidelines suggested that drug resistance testing should be considered based on the patient’s viral load before restarting treatment. If the patient’s viral load is high [>1000 (DHHS, 2024) or 200 (EACS, 2024) copies/mL], a drug resistance test is recommended to select the appropriate ART. Based on our results, we suggest a drug resistance test for unplanned drug interruption patients before reinitiating if VL > 1,000 copies/mL, regardless of their interruption time. For patients with longer interruption times, the patients can detect the drug resistance results through the next-generation sequencing (NGS) method.

Numerous studies (Iarikov et al., 2010; Kalmar et al., 2012) have demonstrated that drug resistance mutations and drug resistance rates would decrease with increasing interruption time. However, our study found that the decline in drug resistance was not as significant as expected. The risk of drug resistance in patients with drug interruption times of 6–12 months and 12–24 months was not statistically different from that in patients with interruption times of 1–6 months. Patients who discontinued ART for more than 24 months exhibited a significantly lower drug resistance rate compared to those who discontinued for 1–6 months. The possible explanation is that NNRTI mutations account for a large proportion of drug-resistant patients in China (Chen et al., 2023), and NNRTI resistance mutation sites (such as K103N) (Kühnert et al., 2018) may persist for several years after drug deletion. Therefore, no significant difference in drug resistance rates was observed between the 1–6 months, 6–12 months, and 12–24 months groups in our study. However, our study found that patients who received ART for 12–24 months before interruption had a lower risk of drug resistance compared to those with ART for 3–12 months. Patients undergoing ART have a higher risk of developing drug resistance as treatment time increases, but this pattern may not apply to patients who have stopped ART. There were three possible explanations for the differences. First, long-term treatment results in better viral suppression and a reduced likelihood of drug resistance mutations. Patients with shorter treatment durations (3–12 months) may not have achieved a stable long-term viral suppression state. Once treatment is interrupted, their viral load is more likely to rebound rapidly (Li et al., 2022), increasing the risk of drug resistance mutations. Secondly, patients who have received ART for 12–24 months may have had their treatment regimens adjusted to use drug combinations with higher resistance barriers, while those treated for 3–12 months are likely still on their initial regimens, which may have lower resistance barriers (Cao et al., 2018). In the case of poor adherence, the risk of drug resistance is higher. Finally, treatment adherence improves with long-term therapy. Patients who have undergone 12–24 months of treatment generally have better adherence than those treated for 3–12 months (Hines et al., 2019). Good adherence helps maintain drug concentrations, thereby reducing the chances of drug resistance mutations. It should be noted that the actual situation may vary due to individual differences, treatment regimens, adherence, and other factors, and needs verification in future studies.

This study included the largest number of HIV drug withdrawal population ever, sourced from two cross-sectional studies and two cohort studies. However, our study also has some limitations. First, most of the drug withdrawal populations came from cross-sectional studies, which inevitably have problems such as recall bias and selection bias. Moreover, cross-sectional studies can only capture the characteristics of patients at a specific time point of drug interruption and do not adequately describe the subsequent changes in drug resistance mutations. Second, numerous unplanned treatment interruptions (Sun et al., 2020; Sun et al., 2021) among PLWH occurred in China during the COVID-19 pandemic, yet our studies were completed prior to the pandemic. The immunity, viral load, drug resistance, and ART reinitiate of withdrawal patients during the COVID-19 pandemic remain unknown. Therefore, surveys of HIV drug-withdrawal patients should be conducted to evaluate their situations post-pandemic.

In conclusion, our study, which included the largest sample of HIV drug-withdrawal patients to date, revealed a drug resistance rate of 19.6% (83/424). These findings highlight the importance of conducting drug resistance tests for patients with unplanned interruptions before reinitiating ART, especially if the viral load exceeds 1,000 copies/mL. Both the HIV resistance rate and the frequency of patients harboring ≥2 drug resistance mutations decreased significantly in the longer interruption time group. Viral load, drug interruption time, and ART duration before drug interruption were considerably correlated with HIV resistance in PLWH.

## Data Availability

The datasets presented in this study can be found in online repositories. The names of the repository/repositories and accession number(s) can be found in the article/Supplementary material.

## Footnotes

^1^ https://hivdb.stanford.edu/
