# Supplementary material for: HIV drug resistance and its associated factors among patients during interruption of antiretroviral therapy in China
Source: Front Microbiol. 2025 Jul 11;16:1617795. doi: 10.3389/fmicb.2025.1617795 (PMC12289575; doi:10.3389/fmicb.2025.1617795)
Supplement: Supplementary file 1 [file Data_Sheet_1.docx]

Supplementary Table 1. Evaluation of the representativeness of 424 and 596 patients compared to the original 999 HIV patients with antiretroviral treatment Interruption in this study.

| Variable | Overall (%) | Overall (%) | Overall (%) | P |
| --- | --- | --- | --- | --- |
| N | 999 | 595 | 424 |  |
| Age (median [IQR], years) | 41.2 (32.1-52.5) | 40.0 (33.0-50.5) | 40.0 (33.0-51.0) | 0.59 |
| Gender = Female (%) | 336 (33.6) | 192 (32.3) | 149 (35.1) | 0.63 |
| Ethnic = Han (%) | 499 (49.9) | 300 (50.4) | 210 (49.5) | 0.96 |
| Education |  |  |  | 0.09 |
| Primary school or below | 672 (67.3) | 397 (66.7) | 305 (71.9) |  |
| Junior high school or above | 314 (31.4) | 196 (33.0) | 117 (27.6) |  |
| Missing | 13 (1.3) | 2 (0.3) | 2 (0.5) |  |
| Residence registration |  |  |  | 0.03 |
| Rural areas | 762 (76.3) | 461 (77.5) | 351 (82.8) |  |
| Urban areas | 142 (14.2) | 74 (12.4) | 51 (12.0) |  |
| Missing | 95 (9.5) | 60 (10.1) | 22 (5.2) |  |
| Job |  |  |  | <0.01 |
| Farmer | 736 (73.7) | 418 (70.3) | 325 (76.6) |  |
| Others | 207 (20.7) | 149 (25.0) | 99 (23.4) |  |
| Missing | 56 (5.6) | 28 (4.7) | 0 |  |
| Marriage |  |  |  | <0.01 |
| Married or cohabiting | 692 (69.3) | 402 (67.6) | 310 (73.1) |  |
| Unmarried | 161 (16.1) | 95 (15.9) | 70 (16.5) |  |
| Other | 106 (10.6) | 69 (11.6) | 44 (10.4) |  |
| Missing | 40 (4.0) | 29 (4.9) | 0 |  |
| Route |  |  |  | 0.33 |
| Sexual transmission | 453 (45.3) | 289 (48.6) | 183 (43.2) |  |
| Drug abuse | 376 (37.6) | 222 (37.3) | 165 (38.9) |  |
| Other | 170 (17.1) | 84 (14.1) | 76 (17.9) |  |
| Spouse or fixed sexual partner infected with HIV |  |  |  | 0.99 |
| Yes | 281 (28.2) | 165 (27.7) | 121 (28.5) |  |
| No | 287 (28.7) | 169 (28.4) | 120 (28.3) |  |
| No spouse or fixed sexual partner | 216 (21.6) | 133 (22.4) | 95 (22.4) |  |
| Missing | 215 (21.5) | 128 (21.5) | 88 (20.8) |  |
| Economy |  |  |  | 0.86 |
| Poor | 421 (42.1) | 243 (40.8) | 176 (41.5) |  |
| Average economic condition or above | 401 (40.1) | 233 (39.2) | 168 (39.6) |  |
| Missing | 177 (17.8) | 119 (20.0) | 80 (18.9) |  |
| Social Support |  |  |  | 0.77 |
| Always or often | 238 (23.8) | 137 (23.0) | 93 (22.0) |  |
| Sometimes or never | 586 (58.7) | 340 (57.2) | 252 (59.4) |  |
| Missing | 175 (17.5) | 118 (19.8) | 79 (18.6) |  |
| Self-satisfied |  |  |  | 0.86 |
| Satisfied | 381 (38.1) | 220 (37.0) | 159 (37.5) |  |
| Unsatisfied | 429 (42.9) | 256 (43.0) | 185 (43.6) |  |
| Missing | 189 (19.0) | 119 (20.0) | 80 (18.9) |  |
| ALCOL |  |  |  | 0.96 |
| Never drink | 313 (31.3) | 180 (30.3) | 130 (30.6) |  |
| Drink | 492 (49.2) | 291 (48.9) | 211 (49.8) |  |
| Missing | 194 (19.6) | 124 (20.8) | 83 (19.6) |  |
